# Supplementary material for: Conditional chemoconnectomics (cCCTomics) as a strategy for efficient and conditional targeting of chemical transmission
Source: eLife. 2024 Apr 30;12:RP91927. doi: 10.7554/eLife.91927 (PMC11060718; doi:10.7554/eLife.91927)
Supplement: Figure 3—source data 3. [file elife-91927-fig3-data3.zip › Figure 3 source data3 part 2.zip!/Figure 3-Source data 3-part2/1053f2-1-CRISPResso_Report_y3hlioj1.zip!/CRISPResso_on_1053f2_2/9.Alleles_frequency_table_around_sgRNA_tggtcctgaccatctacatt.pdf]

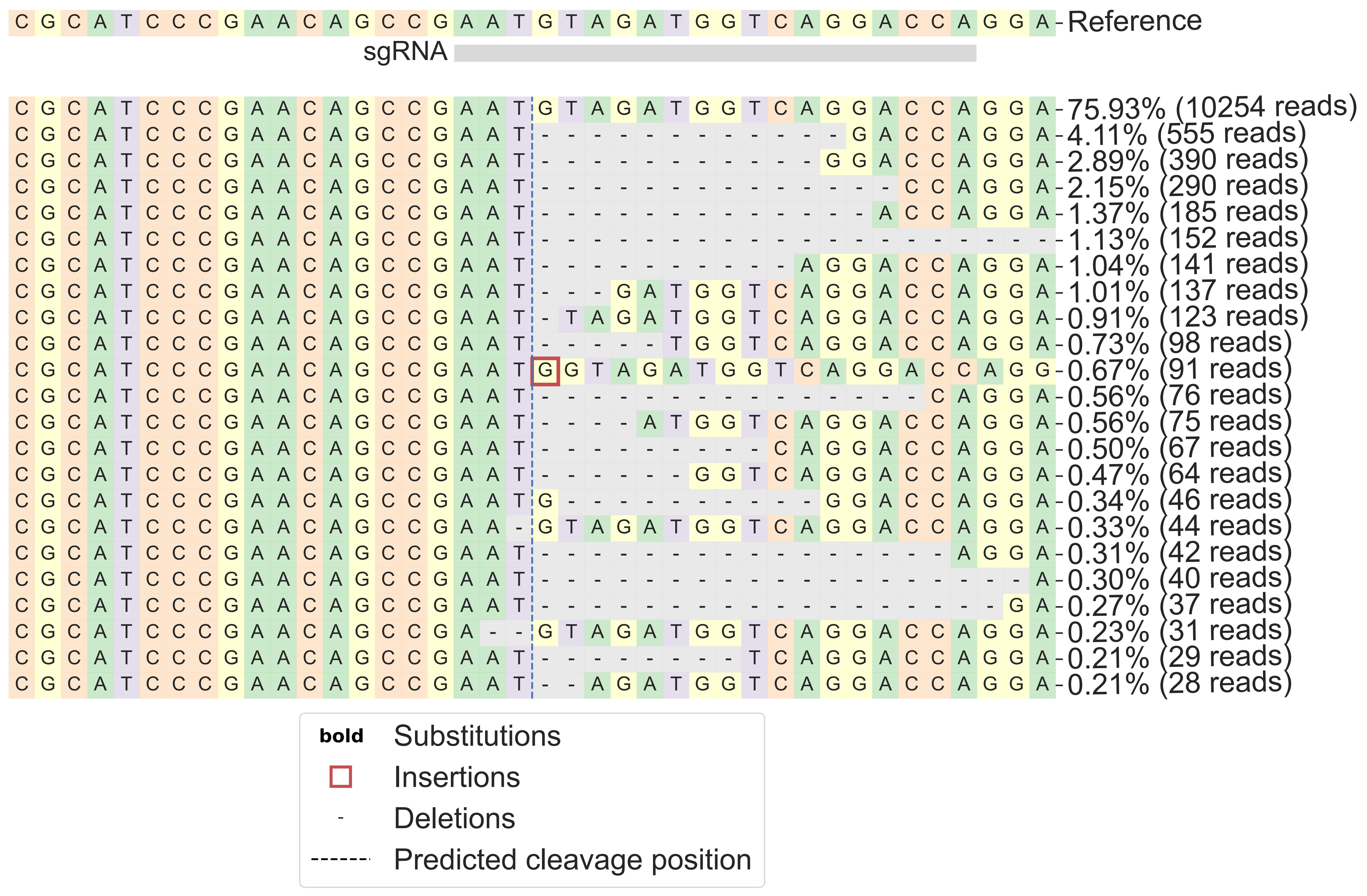
CGCATCCCGAACAGCCGAATGTAGATGGTCAGGACCAGGA Reference
sgRNA
CGCATCCCGAACAGCCGAATGTAGATGGTCAGGACCAGGA 75.93% (10254 reads)
CGCATCCCGAACAGCCGAAT------------GACCAGGA 4.11% (555 reads)
CGCATCCCGAACAGCCGAAT-----------GGACCAGGA 2.89% (390 reads)
CGCATCCCGAACAGCCGAAT--------------CCAGGA 2.15% (290 reads)
CGCATCCCGAACAGCCGAAT-------------ACCAGGA 1.37% (185 reads)
CGCATCCCGAACAGCCGAAT-------------------- 1.13% (152 reads)
CGCATCCCGAACAGCCGAAT----------AGGACCAGGA 1.04% (141 reads)
CGCATCCCGAACAGCCGAAT---GATGGTCAGGACCAGGA 1.01% (137 reads)
CGCATCCCGAACAGCCGAAT-TAGATGGTCAGGACCAGGA 0.91% (123 reads)
CGCATCCCGAACAGCCGAAT-----TGGTCAGGACCAGGA 0.73% (98 reads)
CGCATCCCGAACAGCCGAATGGTAGATGGTCAGGACCAGG 0.67% (91 reads)
CGCATCCCGAACAGCCGAAT---------------CAGGA 0.56% (76 reads)
CGCATCCCGAACAGCCGAAT----ATGGTCAGGACCAGGA 0.56% (75 reads)
CGCATCCCGAACAGCCGAAT---------CAGGACCAGGA 0.50% (67 reads)
CGCATCCCGAACAGCCGAAT------GGTCAGGACCAGGA 0.47% (64 reads)
CGCATCCCGAACAGCCGAATG----------GGACCAGGA 0.34% (46 reads)
CGCATCCCGAACAGCCGAA-GTAGATGGTCAGGACCAGGA 0.33% (44 reads)
CGCATCCCGAACAGCCGAAT----------------AGGA 0.31% (42 reads)
CGCATCCCGAACAGCCGAAT-------------------A 0.30% (40 reads)
CGCATCCCGAACAGCCGAAT------------------GA 0.27% (37 reads)
CGCATCCCGAACAGCCGA--GTAGATGGTCAGGACCAGGA 0.23% (31 reads)
CGCATCCCGAACAGCCGAAT--------TCAGGACCAGGA 0.21% (29 reads)
CGCATCCCGAACAGCCGAAT--AGATGGTCAGGACCAGGA 0.21% (28 reads)
bold Substitutions
Insertions
Deletions
Predicted cleavage position
